# Genome Sequence of a Novel Enterococcus faecalis Sequence Type 922 Strain Isolated from a Door Handle in the Intensive Care Unit of a District Hospital in Durban, South Africa

**DOI:** 10.1128/MRA.00582-19

**Published:** 2019-08-29

**Authors:** Christiana O. Shobo, Daniel G. Amoako, Mushal Allam, Arshad Ismail, Sabiha Y. Essack, Linda A. Bester

**Affiliations:** a Antimicrobial Research Unit, College of Health Sciences, University of KwaZulu-Natal, Durban, South Africa; b School of Laboratory Medicine and Medical Science, Department of Medical Microbiology, University of KwaZulu-Natal, Durban, South Africa; c Biomedical Resource Unit, School of Laboratory Medicine and Medical Sciences, College of Health Sciences, University of KwaZulu-Natal, Durban, South Africa; d Infection Genomics and Applied Bioinformatics Division, Antimicrobial Research Unit, College of Health Sciences, University of KwaZulu-Natal, Durban, South Africa; e Sequencing Core Facility, National Institute for Communicable Diseases, National Health Laboratory Service, Johannesburg, South Africa; University of Maryland School of Medicine

## Abstract

Herein, we highlight the genome sequence of a novel Enterococcus faecalis sequence type 922 (ST922) strain isolated in South Africa. The 3,564,442-bp genome harbored defense systems, a resistome, a virulome, and genetic support, which is of importance to the control of hospital-acquired infections. The genomics of Enterococcus faecalis yields greater understanding into its pathogenesis.

## ANNOUNCEMENT

Enterococcus faecalis is one of the leading causes of hospital-acquired infections (HAIs) (1). Patients acquire HAIs from contaminated hospital environments, including inanimate objects and direct or indirect contact with the hands of health care workers (HCWs) (2, 3). Here, we present the emergence of sequence type 922 (ST922), a novel E. faecalis sequence type found in strain 2SIL2, isolated from a door handle in the intensive care unit of a district hospital in Durban, South Africa. Ethical approval was granted by the Biomedical Research Ethics Committee (BREC) of the University of KwaZulu-Natal, reference BE606/16.

The 2SIL2 strain was isolated and confirmed as Enterococcus faecalis using *Enterococcus* selective agar base (Sigma-Aldrich, St. Louis, MO, USA) and an API 20 strep kit (bioMérieux, Marcy-l’Etoile, France). It was streaked onto a tryptic soy agar (TSA) (Sigma-Aldrich) plate and incubated at 37°C for 24 h. Following incubation, genomic DNA was extracted from 1 CFU of a pure culture of the isolate using the GenElute bacterial genomic DNA kit (Sigma-Aldrich). A paired-end library (2 × 300 bp) was prepared using an Illumina Nextera XT DNA sample preparation kit and sequenced on a MiSeq machine (Illumina, San Diego, CA, USA). The generated sequenced reads (2,003,064 reads) were quality assessed and trimmed using the next-generation sequencing (NGS) core tools in the CLC Genomics Workbench version 11.0.1 (CLC bio/Qiagen, Aarhus, Denmark). Default parameters were used for all software unless otherwise specified. The genome was *de novo* assembled using SPAdes version 3.11 (4), and 927 contigs (99× coverage) were obtained, the largest being 59,734 bp and having an *N*_50_ value of 8,774 bp. The CheckM tool version 0.9.7 (5) was used with lineage-specific marker sets from other genetically well-characterized closely related E. faecalis strains to verify that the sequence reads were not from mixed species. The NCBI Prokaryotic Genome Annotation Pipeline (PGAP) version 4.3 (available at https://www.ncbi.nlm.nih.gov/) and RAST Server version 2.0 (available at http://rast.nmpdr.org) were used for annotation.

The genome features were as follows: genome size, 3,564,442 bp; GC content, 36.80%; total coding sequences (CDS), 4,101; number of coding genes, 3,920; number of RNA genes, 57; number of rRNAs, 3; and number of tRNAs, 49. The novel sequence type was defined as ST922 by the Enterococcus faecalis MLST database (https://pubmlst.org/efaecalis/). BURST algorithmic analysis identified ST93 (a double-locus variant) as the potential parent ancestry to the clone. With the CRISPRCasFinder (6) at default settings, 5 putative CRISPR arrays were identified on contigs 45, 46, 47, 48, and 129, with 2 associated Cas clusters on contigs 48 and 129 of the genome. The antibiotic resistome included those for aminoglycosides [*aph(3′)-III*, *ant(6)-Ia*, *str*], macrolide-lincosamide-streptogramin [*lsa(A)*, *erm(B)*, and *lnu(G)*], oxazolidinone (*optrA*), phenicol (*cat, fexA*), tetracycline [*tet*(M), *tet*(L)], and trimethoprim (*dfrG* and *dfrK*) (according to the ResFinder tool Web platform, version 3.1) (7).

PlasmidFinder version 1.3 (8) identified three plasmid replicons (rep7, rep9, and rep11). The PHAge Search Tool (PHAST) (9) detected two intact phages (*Entero_phiFL3A_NC_013648* and *Paenib_Xenia_NC_028837*). The insertion sequences in the genomes (IS*3*, IS*66*, IS*1634*, and IS*701*) were predicted by BLAST searches against contigs on the ISfinder database (10). The GoSeqIt VirulenceFinder database version 2.0 discovered the following putative virulence determinants (using a threshold identity of ≥95% and a minimum length of 60%): aggregation substance (*agg*), pheromone precursor lipoproteins (*cad*, *camE*, *cCF10*, and *cOB1*), endocarditis- and bioﬁlm-associated pili (*ebpA*, *ebpB*, and *ebpC*), endocarditis antigen A (*efaAfs*), enterococcal leucine-rich internalin-like protein A (*elrA*), gelatinase (*gelE*), hyaluronidase (*hylA* and *hylB*), sortase A (*srtA*), and thiolperoxidase (*tpx*), potentially contributing its ability to adhere to surfaces, invade the immune system, colonize, and cause harmful effects to the host (11). The genomics of Enterococcus faecalis highlights the need for effective infection control systems in health care facilities to prevent HAIs.

### Data availability.

This whole-genome sequence project has been deposited in DDBJ/ENA/GenBank with the BioProject and BioSample numbers PRJNA523601 and SAMN10984490 under the accession number SIYF00000000. The described version is SIYF01000000. The raw reads have been submitted to the SRA (accession number SRR9021436).
